# Investigation of the Effect of Deprivation on the Burden and Management of Venous Leg Ulcers: A Cohort Study Using the THIN Database

**DOI:** 10.1371/journal.pone.0058948

**Published:** 2013-03-19

**Authors:** Emily S. Petherick, Nicky A. Cullum, Kate E. Pickett

**Affiliations:** 1 Born in Bradford, Bradford Institute for Health Research, Bradford, United Kingdom; 2 School of Nursing, Midwifery and Social Work, University of Manchester, Manchester, United Kingdom; 3 Department of Health Sciences, University of York, York, United Kingdom; 4 Hull York Medical School, University of York, York, United Kingdom; California Pacific Medicial Center Research Institute, United States of America

## Abstract

**Background:**

There has been limited examination of the contribution of socio-economic factors to the development of leg ulcers, despite the social patterning of many underlying risk factors. No previous studies were found that examined social patterns in the quality of treatment received by patients with leg ulcers.

**Methods:**

Using The Health Improvement Network (THIN) database we identified a cohort of over 14000 patients with a diagnosis of venous leg ulceration, prospectively recorded between the years 2001 and 2006, with linked area-level socio-economic information (Townsend deprivation quintile). We assessed socio-economic differences in the incidence and prevalence of leg ulcers using negative binomial regression. Socio-economic differences in two key areas of guideline recommended leg ulcer management, arterial Doppler assessment and compression bandaging, were assessed using multilevel regression.

**Results:**

The risk of incident venous leg ulceration increased for patients living in areas of higher deprivation, even after adjustment for known risk factors age and gender. Overall reported rates of Doppler assessment and provision of compression therapy were low, with less than sixteen per cent of patients having a database record of receiving these recommended diagnostic and treatment options. Patients diagnosed with incident venous leg ulcers living in the most deprived areas were less likely to receive the recommended Doppler-aided assessment for peripheral vascular disease than patients living in the least deprived areas (odds ratio 0.43, 95% confidence interval 0.24–0.78). Documented provision of compression therapy did not vary with deprivation.

**Conclusions:**

A socio-economic gradient in venous leg ulcer disease was observed. The overall rates of people with venous leg ulcers who were documented as receiving guideline recommended care (2001–2006) were low. Reported use of Doppler ultrasound assessment was negatively associated with socio-economic status. These findings suggest that the inequalities experienced by leg ulcer patients may be exacerbated by reduced access to guideline-based management.

## Introduction

Prevalence studies, undertaken nationally and internationally, have produced consistent estimates that one per cent of the population has chronic leg ulceration; with approximately 20% of these people having an open ulcer at any point in time [1]. Prevalence rates of leg ulceration are generally higher in women than men and increase with age [2]. Studies suggest that the majority of leg ulcers are associated with venous disease (estimates range from 40 to 80%), but other risk factors include immobility, obesity, trauma, arterial disease, vasculitis, diabetes and neoplasia [2]–[4] Regardless of the pathology, all leg ulcers represent a failure of the underlying vessels to effectively transport blood to and from the tissues of the lower limbs. Most leg ulcers are slow to heal, with up to 68 per cent of cases recurring within a two year period [5]. Leg ulcers typically produce exudate, reduce mobility, smell and can be incapacitating and socially isolating [6].

There have been few evaluations of the relationship between socio-economic factors and leg ulcer disease burden and these evaluations do not provide consistent evidence of a socio-economic gradient in leg ulcer rates [7], [8]. There are several reasons why socio-economic factors may be relevant. First, some of the risk factors associated with venous disease severity, for example obesity, [9] have a social gradient, as the prevalence of obesity is highest among women with low socio-economic status [10]. Furthermore, it is known that the risks of both venous insufficiency and peripheral arterial disease increase with cigarette smoking [11], [12] which also has a social gradient in both individual and area based measures of socio-economic status. Stress has been shown to influence wound healing [13] and has been associated with individual socio-economic status [14]. There is also abundant evidence that socio-economic factors, related to the patient and the area in which the patient resides, influence the nature of, and access to, health care [15]–[18]. The social determinants of health encountered by the individual and the area in which they live and receive treatment may therefore influence many aspects of the development, persistence and recurrence of leg ulcers.

Three UK guidelines for the management and treatment of leg ulcers provide two consistent messages which can be implemented by all practitioners treating patients with leg ulcers, including those in primary care [17]–[19]. First, all patients presenting with leg ulcers should have the arterial circulation of their leg assessed so that significant arterial insufficiency can be excluded as a contributing factor to the ulceration and a contra-indication for compression bandaging. Measurement of the ankle brachial pressure index (ABPI) aided by Doppler ultrasound is the recommended technique for this initial assessment of the arterial supply [17]–[19]. Second, the recommended first line therapy for patients with uncomplicated venous leg ulceration is compression bandaging. This recommendation for the use of compression bandaging is supported by the results of a systematic review [20].

In this cohort study our objective was to analyse potential relationships between socio-economic factors and incidence, prevalence and the two aspects of guideline recommended management, in adults with venous leg ulcers consulting in general practice between 2001 and 2006, using a national care primary care dataset.

## Materials and Methods

### Ethical Statement

Ethical approval to conduct the study using the THIN database was granted by the Cambridgeshire 4 Research Ethics Committee, reference number 08/H0305/21.

### Data Source

We examined individual level patient data from primary care using The Health Improvement Network (THIN) database of anonymous patient records which were prospectively collected from general practice. This database is similar to the general practice research database (GPRD), a validated primary care database, to which over half of all practices contributing to the THIN database also provide data [21]. The THIN database was chosen in preference to the GPRD as the THIN database contained an area based measure of socio-economic status in contrast to the GPRD, which did not at the time this study was conducted.

The THIN database has been shown to be representative of the UK general practice population [22]. Furthermore, consultation and prescription rates recorded in the THIN database have been shown to be comparable with published estimates [23].

### Study Sample

Two cohorts of venous leg ulcer patients were identified in the THIN database. The first cohort comprised people with prevalent venous leg ulcers who were aged 18 years or over, had consulted between 2001 and 2006 and had a database record of a diagnosed venous leg ulcer. The second cohort comprised patients with a database record of an incident venous leg ulcer. All cases were identified using a Read code algorithm for venous leg ulcer patients previously developed, and validated in the GPRD, by Margolis *et al* [24]. The inclusion criteria for incident and prevalent venous leg ulcer cases described by Margolis *et al* [24] were adapted to the THIN database to allow for differences between the GPRD and THIN data quality criteria. In the THIN database, data are assumed to be of high quality if they meet an acceptable mortality reporting criterion. Therefore this criterion was used to select data for the current analysis to attempt to replicate the “up to standard” time criterion of the GPRD as used by Margolis *et al* [24]. Replicating the methods undertaken by Margolis *et al* [24], we have only included data that were prospectively reported in the THIN database. Any events occurring prior to practice registration were not examined as they may be subject to recall bias. These same criteria were applied to the denominator data supplied to us by the THIN database administrators. Person-time was calculated for this study by THIN database administrators using the same methods previously used by Margolis *et al* [24]. The starting time was defined as six months after the patient’s first practice visit and the end time as their last database record or the end of the study period if the patient was still registered with a THIN practice. The denominator for prevalence was all patients aged 18 years and over, who had made at least one visit to their general practice clinic in a given year.

The socio-economic information available for each patient in the THIN databases is the Townsend deprivation index quintile, a measure of material deprivation calculated using census data and linked to area of residence. The Townsend deprivation index [26] is calculated using the following domains; unemployment as a percentage of those aged 16 years of over who are economically active, car ownership as a percentage of all households, home ownership as a percentage of all households and overcrowding. It has been used extensively in UK based studies to examine relationships between deprivation and health. These domains were obtained from the 2001 census data and linked to a person’s residence using their postcode data. In order to maintain patient anonymity the Townsend deprivation score is calculated by the THIN database administrators. These raw Townsend scores are then converted into quintile rank for use by external researchers accessing the THIN data. Multiple deprivation quintile data were available for fewer than one hundred patients with a database record of venous leg ulcer in cases where patients had moved residence during the study period. In these cases the most recent value, reflecting their current residential address was used.

Patients may have had leg ulcers prior to their initial registration with a THIN practice. To minimise reporting bias, the investigation of patient management was restricted to the incident cohort, as it was likely that some patients would have been treated prior to the recording of a Read coded diagnosis of venous leg ulceration in THIN. Database records were searched for Read codes indicative of receiving a Doppler assessment or compression bandaging in the thirty days prior to, and in the ninety days post, any diagnosis. A ninety day cut-off was chosen as Margolis *et al* [24] suggest that ‘*three months is a reasonable length of time to allow for a complete evaluation and diagnosis of a person with a leg ulcer*’. It has also been suggested that patients should have repeat ultrasound at three monthly intervals [25].

### Statistical Analyses

Annual incidence density over the entire study period was calculated using the following formula, where person years are defined as the time at risk for development of a leg ulcer from initial registration:
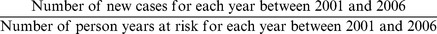



Prevalence was calculated by including all patients aged 18 years and over diagnosed with a venous leg ulcer who had made at least one visit to their general practice clinic in a given year (a baseline ulcer free-period was not required).

Annual period prevalence was calculated using the formula:
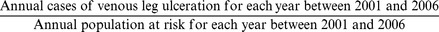



Negative binomial regression models were then used to investigate the relationships between explanatory variables including age, gender, deprivation, study year and rates of venous leg ulcer incidence and prevalence. Negative binomial regression was used in preference to Poisson regression as likelihood ratio tests showed evidence of an over-dispersion of nonzero counts relative to the Poisson distribution [26].

The probability of patients receiving the two recommended interventions (Doppler ultrasound-aided assessment and compression bandaging) was investigated using multilevel logistic regression models. This enabled the relative contribution of explanatory variables at both the patient and practice level to be investigated. Multilevel models were used as patients are clustered within different general practice groups, i.e. patients attending the same practices will be more similar than those attending different practices and failure to account for the clustered nature of the data may lead to biased regression estimates and artificially small confidence intervals [27], [28].

## Results

### Demographics

Throughout the study period of 2001 to 2006, 135 142 89 adult patients registered in a THIN practice contributed 132 671 82 person years of data. Of these patients, 16 500 were identified with a database diagnosis of venous leg ulcer and of these, 14 568 met the criteria for having an incident venous leg ulcer. Greater proportions of both incident and prevalent patients were women, with a mean age greater than 73 years (Table 1). In descriptive analyses, there was no observed socio-economic gradient in the proportion of patients diagnosed with either incident or prevalent leg ulcers by area-level Townsend deprivation quintile.

**Table 1:** Baseline characteristics of the incident and prevalent cohort in the THIN database 2001 to 2006.

Ulcer type	Incident venous leg ulcers	Prevalent venous leg ulcers
N	14,568	16,500
Female, N (%)	9,158 (62.9)	10,307 (62.5)
Mean age (SD), Median, range, in years	73.7 (14.4)	74.3 (14.2)
	77, 18–109	77, 18–109
Townsend deprivation quintile, N (%)		
1 (least deprived)	2802 (19.2)	3172 (19.2)
2	2871 (19.7)	3303 (20.0)
3	3060 (21.0)	3419 (20.7)
4	2796 (19.2)	3153 (19.1)
5 (most deprived)	2065 (14.2)	2375 (14.4)
Missing	974 (6.7)	1078 (6.5)

### Incidence and Prevalence

The independent relationship of area-level Townsend deprivation quintile rank to incidence and prevalence rates was investigated and shown below in table 2. The results for both incidence and prevalence indicated that strong socio-economic gradients exist after adjustment for age and gender, with rates of venous leg ulcers increased with every increase in deprivation quintile rank compared to the least deprived quintile rank.

**Table 2:** Results of negative binomial regression examining the relationship of deprivation quintile rank to venous leg ulcer burden.

Incidence	Unadjusted Incidence Rate Ratio (95% CI) p-value	Adjusted Incidence Rate Ratio (95% CI) p-value*
Townsend deprivation quintile		
1 (less deprived)	1	1
2	1.06 (0.90, 1.24) 0.51	1.08 (1.02,1.14) <0.001
3	1.11 (0.94, 1.30) 0.21	1.24 (1.18, 1.31) <0.001
4	1.10 (0.93, 1.29) 0.25	1.30 (1.23,1.38) <0.001
5 (more deprived)	1.15 (0.97, 1.36) 0.10	1.44 (1.36,1.53) <0.001
p for linear trend		<0.001
**Prevalence**	**Unadjusted Incidence Rate Ratio (95% CI) p-value**	**Adjusted Prevalence Rate Ratio (95% CI) p-value**
Townsend deprivation quintile rank		
1 (less deprived)	1	1
2	1.05 (0.89, 1.24) 0.55	1.10 (1.04, 1.17) <0.001
3	1.06 (0.90, 1.25) 0.47	1.25 (1.18, 1.32) <0.001
4	1.04 (0.89, 1.23) 0.61	1.31 (1.24, 1.39) <0.001
5 (more deprived)	1.12 (0.95, 1.32) 0.20	1.48 (1.39, 1.57) <0.001
p for linear trend		<0.001

* Results adjusted for age, gender and study year.

### Leg Ulcer Management

Reported rates of ABPI measurement by Doppler ultrasound among patients with incident venous leg ulcers were low, with only 1 608/14 568 (11.0%) of patients having a record of ABPI measurement within three months of initial diagnosis. There was considerable variation in the proportion of venous leg ulcer patients recorded as having received the assessment between practices, with rates ranging from zero to 100 per cent of all patients within an individual practice.

The relationship between deprivation and recording of two aspects of venous leg ulcer management was investigated. First, the initial assessment the venous ulcer using ABPI was investigated and is shown in table 3. Secondly the initial provision of compression bandaging was examined and shown below in table 4.

**Table 3:** Results of multilevel logistic regression analysis of Doppler aided ABPI measurement for patients diagnosed with venous leg ulceration.

Individual level variables	Unadjusted Odds Ratio	Adjusted odds Ratio (95% CI)
Age (years)		
18–27	0.10 (0.01, 0.99)	0.10 (0.01, 1.03)
28–37	0.25 (0.07, 0.87)	0.23 (0.06, 0.82)
38–47	0.57 (0.25, 1.32)	0.55 (0.24, 1.29)
48–57	1.10 (0.62, 1.97)	1.03 (0.57, 1.84)
58–67	1.52 (0.95, 2.41)	1.40 (0.88, 2.22)
68–77	1	1
78–87	0.50 (0.34, 0.74)	0.50 (0.34, 0.75)
88–97	0.11 (0.06, 0.23)	0.11 (0.06, 0.23)
98+	0.09 (0.01, 0.94)	0.10 (0.01, 1.01)
Gender		
Male	1	1
Female	0.81 (0.72–0.90)	0.75 (0.55, 1.02)
Index of multiple deprivation quintile		
1 (low deprivation)	1	1
2	0.81 (0.51–1.28)	0.88 (0.56, 1.38)
3	0.72 (0.45–1.15)	0.80 (0.51, 1.26)
4	0.82 (0.50–1.33)	0.88 (0.55, 1.40)
5 (high deprivation)	0.41 (0.23–0.75)	0.43 (0.24, 0.78)
Year of diagnosis		
2001	1	1
2002	1.83 (1.02, 3.26)	1.62 (0.92, 2.83)
2003	2.56 (1.43, 4.54)	2.73 (1.55, 4.80)
2004	3.31 (1.83, 5.98)	3.45 (1.92, 6.18)
2005	5.50 (2.96, 10.23)	4.50 (2.67, 8.98)
2006	5.00 (2.70, 9.27)	4.61 (2.42, 8.45)
**Practice level variable**		
Mean practice list size		
0–4999	0.02 (0.01, 0.27)	0.02 (0.01, 0.28)
5000–9999	0.27 (0.08, 0.94)	0.24 (0.07, 0.81)
10000	0.32 (0.11, 0.95)	0.31 (0.10, 0.90)
15000	0.31 (0.10, 1.03)	0.33 (0.10, 1.06)
20000	1	1
25000	1.24 (0.34, 4.47)	1.20 (0.34, 4.19)
30000	7.58 (1.19, 48.50)	6.56 (1.08, 39.92)
35000	8.57 (1.30, 56.68)	8.70 (1.37, 55.14)

**Table 4:** Results of multilevel logistic regression analysis of compression bandaging provision for patients diagnosed with venous leg ulceration.

Individual level variables	Unadjusted Odds Ratio	Adjusted odds Ratio (95% CI)
Age (years)		
18–27	0.32 (0.09, 1.18)	0.34 (0.09, 0.34)
28–37	0.62 (0.30, 1,27)	0.68 (0.33, 1.41)
38–47	0.40 (0.23, 0.72)	0.44 (0.25, 0.78)
48–57	0.51 (0.34, 0.78)	0.58 (0.38, 0.87)
58–67	0.95 (0.71, 1.28)	0.96 (0.72, 1.30)
68–77	1	1
78–87	1.40 (1.10, 1.79)	1.39 (1.09, 1.78)
88–97	1.12 (0.83, 1.51)	1.10 (0.81, 1.49)
98+	1.30 (0.41, 4.11)	1.27 (0.40, 4.06)
Gender		
Male	1	1
Female	1.34 (1.10, 1.62)	1.20 (0.99, 1.46)
Deprivation quintile		
1 (low deprivation)	1	1
2	1.19 (0.90, 1.56)	1.16 (0.87, 1.53)
3	1.07 (0.82, 1.14)	1.07 (0.81, 1.43)
4	1.12 (0.84, 1.49)	1.11 (0.84, 1.49)
5 (high deprivation)	1.11 (0.80, 1.53)	1.18 (0.85, 1.65)
Year of diagnosis		
2001	1	1
2002	0.87 (0.63, 1.18)	0.88 (0.64, 1.21)
2003	0.70 (0.51, 0.97)	0.71 (0.51, 0.98)
2004	0.93 (0.68, 1.27)	0.92 (0.67, 1.26)
2005	1.22 (0.89, 1.66)	1.18 (0.86. 1.62)
2006	1.24 (0.91, 1.71)	1.20 (0.87, 1.66)
**Practice level variable**		
Mean practice list size		
0–4999	0.66 (0.16, 2.75)	0.69 (0.16, 2.97)
5000–9999	0.94 (0.41, 2.15)	0.93 (0.40, 2.17)
10000	1.06 (0.50, 2.24)	1.08 (0.50, 2.33)
15000	1.16 (0.51, 2.62)	1.17 (0.51, 2.70)
20000	1	1
25000	1.39 (0.56, 3.43)	1.38 (0.55, 3.48)
30000	0.38 (0.09, 1.54)	0.37 (0.09, 1.53)
35000	0.59 (0.14, 2.41)	0.60 (0.14, 2,54)

Older venous leg ulcer patients (78–87 years) were less likely to receive Doppler-aided assessment than those aged 68–77 years (odds ratio 0.50, 95% confidence interval 0.34 to 0.75) as were patients living in areas of high deprivation (odds ratio 0.43, 95% confidence interval 0.24 to 0.78) compared to those living in areas of low deprivation. The odds of receiving Doppler assessment increased with year of diagnosis from 2003 onwards, compared to 2001, with an odds ratio of 4.61 (95% confidence interval 2.42 to 8.45) by 2006. Practice size also made both negative and positive contributions to the odds of receiving Doppler ultrasound assessment. Compared with a practice size of 20 000 registered patients, smaller practices were associated with reduced odds of receiving Doppler assessment, whereas people attending larger practices had increased odds of receiving Doppler assessment of their venous leg ulcer. Although women had reduced odds of receiving Doppler assessment than men (odds ratio 0.75, 95% confidence interval 0.55 to 1.02) this difference was no longer statistically significant after adjustment for other confounders. The practice level variable was shown to contribute to over 30 per cent of the variation in the results.

A record of the prescription or application of compression bandaging was located for 2 967/14 568 (20.4%) patients. There was a high degree of practice-level variation in the proportion of people documented as having venous leg ulcers also documented as receiving compression therapy (0–100%).

Older people (78 to 87 years) were more likely to receive compression than people aged 68 to 77 years (odds ratio 1.39, 95% confidence interval 1.09 to 1.78). Although the odds of receiving compression were also lower in people younger than 67 years and also older than 88 years, these differences were not statistically significant. Other variables examined including gender, deprivation, year of diagnosis and practice size were not significantly associated with the odds of receiving compression therapy. The practice level variable was again shown to contribute to over 30 per cent of the variation in the results.

## Discussion

The first aim of this study was to determine whether socio-economic factors were associated with the incidence and prevalence of venous leg ulceration and we found a 10% increase in risk with each area-level deprivation quintile, from lowest to highest deprivation, independent of age, gender and year of study. Women and older people were also at increased risk. The second aim was to examine socio-economic differences between patients in the initial diagnosis and management of leg ulceration. UK guidelines recommend Doppler ultrasound-aided ABPI measurement to guide appropriate management [17]–[19] but evidence of this was rarely documented. The relevant clinical practice guidelines evaluated were widely disseminated throughout the NHS by the National Institute for Health and Clinical Excellence, were freely available online and were actively disseminated within the National Health Service by specialist teams in Scotland. Multilevel logistic regression showed that patients living in areas of highest material deprivation were less likely to have a documented Doppler-aided ABPI measurement, although the odds of receiving ABPI improved over time. The population most likely to develop leg ulcers, those living in areas of high deprivation and older people, were the least likely to receive recommended diagnostic assessment which may exacerbate existing health inequalities. With the exception of compression therapy for venous leg ulceration [20], few therapies for leg ulceration have high quality evidence of effectiveness. The odds of receiving compression therapy were not significantly related to deprivation. However, increasing patient age and more recent year of diagnosis were both shown to be associated with higher odds of receiving compression therapy suggesting that use of this therapy is increasing over time.

This study has some weaknesses. First, none of the codes used to identify venous leg ulcer patients in the THIN database could be specifically validated in this study, however, over half of the practices that contribute to the THIN database also contribute to the GPRD [21], and the same diagnostic codes for venous leg ulceration have been validated in the GPRD [24]. The results presented are based on recorded consultations and we cannot eliminate the possibility of incomplete reporting of patient’s diagnoses and management contributing to the observed results. Although most leg ulcers are diagnosed and treated by GPs, there may be some patients who choose to self-treat their leg ulcers. Given that there is no charge at the point of prescription for persons in the UK aged 65 years or over it is unlikely that there is a systematic bias in the socioeconomic status of patients choosing to self-treat their venous leg ulcers. We have been unable to adjust for both smoking and obesity in these analyses and acknowledges that these variables may explain some of the differences in leg ulcer rates by deprivation as both smoking and obesity rates are associated with area level deprivation. It was not possible to measure individual-level socioeconomic status in this study, instead area-level measures of deprivation for each patient’s residential postcode were used as a proxy individual measure. Small area measures of socioeconomic status have been considered reasonable proxies in epidemiological studies [29], nevertheless they do not allow any disentangling of the effects of individual socioeconomic status versus any effect of area level social context, and are only good proxy measures to the extent that postcodes are relatively homogeneous for socioeconomic status. This study used Townsend deprivation quintiles based on indicators from the 2001 census, applied to the residential areas in which patients lived in 2006. Two problems arise - first, the deprivation score might not accurately reflect deprivation over time, and second, there is a possibility that the development of a leg ulcer may cause a change in residence to a more deprived area. The latter is unlikely to have been a major factor since fewer than 100 subjects had multiple deprivation quintile data. The majority of patients were also aged over 65 years and rates of internal migration within the UK have been shown to be low in this age group [30].

Key strengths of our study include the size and representativeness of the sample; this is largest study yet undertaken to evaluate the effect of socioeconomic factors on both the burden and management of leg ulceration and estimates were not subject to recall or non-responder bias, which may have been present in previously published surveys.

Incidence and prevalence rates of venous leg ulceration are broadly comparable with previous studies. Crude prevalence rates of 0.08–0.12 per 100 persons for women and 0.05 to 0.08 per 100 persons for men observed in this analysis are within the bounds of those reported in a systematic review by Graham *et al.* [1]. Rates of incidence observed in the current study are lower than those reported by Margolis *et al.* [24], but in our study are obtained from a general adult rather than an elderly population. These comparisons are, however, of non-standardised rates and must be interpreted with caution. This study also confirms those of others showing a social patterning of prevalent leg ulcers [31], [8], and shows that incidence is also related to socioeconomic status, suggestive of a causal pathway. In the present study we have strengthened the external validity and generalisability of these earlier findings by the use of nationally representative data with a large sample size.

We were unable, in this database, to explore a reported association between social class and ulcer duration identified by Callam et al. [7], as repeat visits for chronic conditions are not routinely recorded in the database unless they are associated with a change of management. No previous studies were found that examined the influence of socio-economic factors on the management of leg ulceration, however the results of studies conducted in primary care have found similarly low levels of reported use of Doppler ultrasound-aided assessment. Hickie *et al* [31] found that only 8% of all professionals in primary care reported using Doppler ultrasound. Studies have also shown that many practitioners working with leg ulcer patients do not have confidence in their ability to conduct ABPI assessment using Doppler ultrasound [32], [33], further it remains unclear if all practices have access to Doppler ultrasound despite the equipment required to undertake this test being relatively inexpensive. Despite these deficiencies in practice, results from the THIN database suggest that more patients are receiving guideline recommended venous leg ulcer care over time, indicating that these guideline recommendations are being implemented, albeit slowly. Other studies examining usage rates of compression have reported higher rates ranging from 50 to 90% of all patients [32], [34], [35] compared to this study, where they ranged from 0 to 100% of all venous leg ulcer patients within individual practices. With the exception of one study which reviewed the case notes of 66 patients [35], other studies were based on surveys of health professionals with poor response rates [32]. The reported usage rates from those studies may paint an overoptimistic picture of the realities of clinical practice. It remains unclear whether lower usage rates of compression therapy observed in our study are a reflection of referral practices where treatments provided by community nurses may not be recorded in the THIN database. Whilst there may be some reporting differences between different practitioner groups in primary care these differences are unlikely to explain the social gradients in reported management observed in the current analyses.

The results of the current study provide yet more evidence of inequalities in health that have the potential to be either ameliorated or exacerbated by contact with primary care providers. The results of the current study suggest that better adherence to leg ulcer management guidelines would have the potential to eliminate the inequalities observed in the development of venous leg ulcers [17]–[19]. Initiatives such as the Quality and Outcomes Framework [36] provide evidence that incentives for primary care providers to increase the high quality care provision to all members of society may contribute to a reduction in health inequalities.

Acknowledgements: The authors would like to acknowledge the statistical advice provided by Professor J Martin Bland as well as feedback on earlier drafts provided by Professor Ian Watt.

This article presents independent research commissioned by the National Institute for Health Research (NIHR) when ESP was funded by the Personal Awards Scheme (Researcher Development Award). Professor N Cullum is an NIHR Senior Investigator. The views expressed in this publication are those of the author(s) and not necessarily those of the NHS, the NIHR or the Department of Health.
